# Coexistence of Lichen Sclerosus and Morphea in a Single Vulvar Lesion

**DOI:** 10.7759/cureus.77303

**Published:** 2025-01-11

**Authors:** Kazuyoshi Iijima, Yoshihito Mima

**Affiliations:** 1 Department of Dermatology, Teikyo Mizonokuchi Hospital, Kanagawa, JPN; 2 Department of Dermatology, Tokyo Metropolitan Police Hospital, Tokyo, JPN

**Keywords:** coexistence, lichen sclerosus, lichen sclerosus et atrophicus, localized scleroderma, morphea

## Abstract

Lichen sclerosus (LS) and morphea are chronic inflammatory disorders with overlapping clinical and histopathological features, often complicating diagnosis. While the co-occurrence of LS and morphea as distinct lesions in the same patient is well documented, their overlap within a single lesion is rare, especially in genital regions. Herein, we present a case of a 60-year-old female with coexisting LS and morphea in the genital region, exhibiting features of LS while demonstrating histological characteristics of both. This case underscores the diagnostic challenges and provides further support for the possibility that LS and morphea may represent varying manifestations within a shared disease spectrum.

## Introduction

Lichen sclerosus (LS), also known as lichen sclerosus et atrophicus (LSA), is a chronic inflammatory dermatosis primarily affecting the genital region, presenting as white, keratotic plaques with atrophic changes [1]. In contrast, morphea, also known as localized scleroderma, is a rare inflammatory connective tissue disorder characterized in its active phase by violaceous erythematous plaques. These lesions gradually evolve into sclerotic and atrophic plaques with well-demarcated borders, which may appear white or yellowish in chronic cases [2]. LS predominantly involves the superficial dermis, leading to atrophic and inflammatory changes [1]. On the other hand, morphea typically affects the dermis and subcutaneous layers, causing fibrosis and tissue remodeling [2]. Although LS predominantly involves the anogenital region, reports of its coexistence with morphea within a single lesion are largely confined to the extragenital areas. Herein, we present a rare case of overlapping LS and morphea in a single vulvar lesion.

## Case presentation

**Figure 1:**
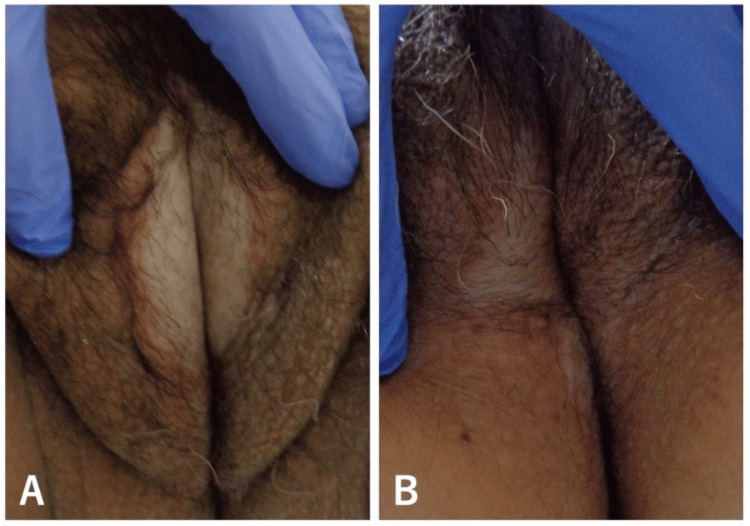
(A) Sclerotic white plaques on the labia majora, with intermixed white papules. (B) Comparable sclerotic plaques were also observed in the perianal region. Original image by the authors.

A 60-year-old female presented with pruritus and painful white sclerotic plaques on the vulvar and perianal regions. She initially noticed white discoloration in these areas one year prior, followed by the onset of pruritus and pain six months later. Despite treatment with topical corticosteroids, her symptoms progressively worsened, leading to her referral for further evaluation. The patient's medical history included depression, recurrent urinary tract infections, and thyroid cancer, which was treated with a thyroidectomy 30 years ago. Since then, she has been on levothyroxine sodium with stable thyroid hormone levels. Over the past year, she experienced three episodes of urinary tract infection, each successfully managed with a one-week course of cephalexin, resulting in full recovery. Her regular medications included levothyroxine sodium and paroxetine hydrochloride, and there was no significant family history. Physical examination revealed well-demarcated, sclerotic white plaques on the labia majora, along with scattered white papules. Similar lesions were noted in the perianal region (Figure 1). No other skin manifestations suggestive of morphea, such as violaceous plaques or generalized sclerotic changes, were identified. Systemic symptoms were not observed.

**Figure 2:**
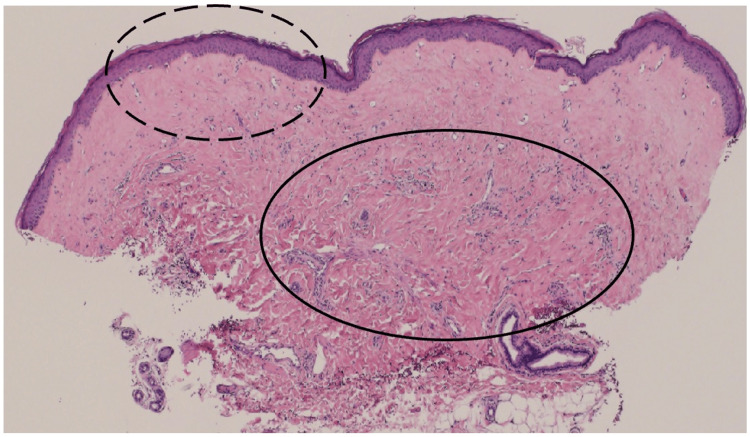
Low-power view of the biopsy specimen from the labia majora. Original image by the authors.

**Figure 3:**
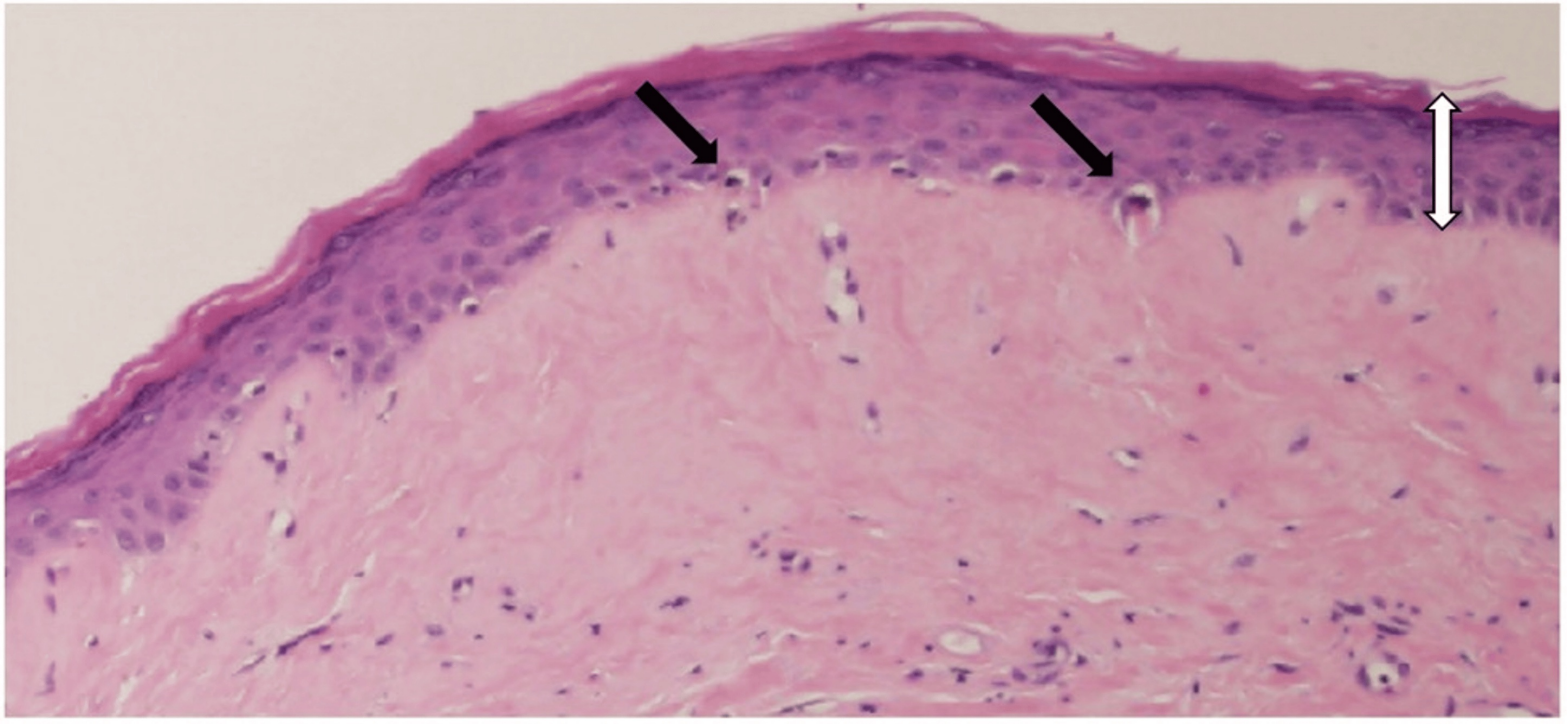
Higher magnification of the dotted circular area in Figure 2, demonstrating a flattened epidermis (white arrow), focal liquefactive degeneration of the basal layer (black arrows), and homogenized collagen fibers in the superficial dermis. Original image by the authors.

**Figure 4:**
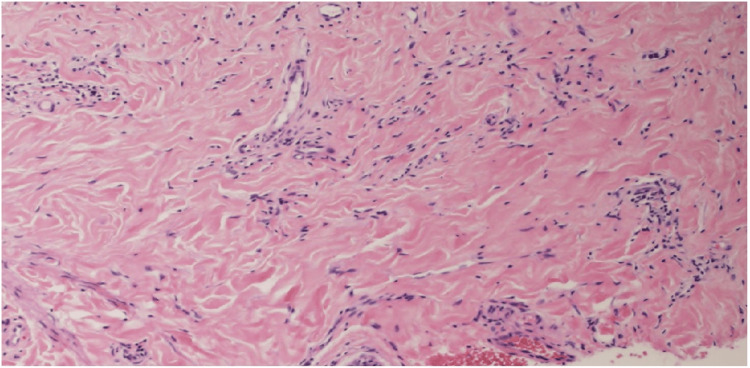
Higher magnification of the circled area in Figure 2, demonstrating pronounced thickened and inflated collagen bundles in the deeper dermis. Original image by the authors.

Laboratory testing revealed mild leukocytosis (10,300/μL; reference range, 4000-10,000/μL) and elevated C-reactive protein (CRP, 0.76 mg/dL; reference range, 0.00-0.30 mg/dL). Liver and renal function tests, immunoglobulin levels, and autoantibody screening (including Sjögren's syndrome-related antigen A (SS-A), Sjögren's syndrome-related antigen B (SS-B), scleroderma antibody (SCL), histidyl-tRNA synthetase antibody (Jo-1), single-stranded DNA antibody (ss-DNA), double-stranded DNA antibody (ds-DNA), and centromere antibodies) were unremarkable. Histopathological analysis of a biopsy from the labia majora revealed mild hyperkeratosis, epidermal atrophy, and liquefactive degeneration of the basal layer (Figures 2, 3). The upper dermis showed homogenized collagen fibers and edema, while the deeper dermis exhibited thickened collagen bundles and inflammatory cell infiltration (Figures 2, 4). No hyalinized collagen band was observed in the superficial dermis.

**Figure 5:**
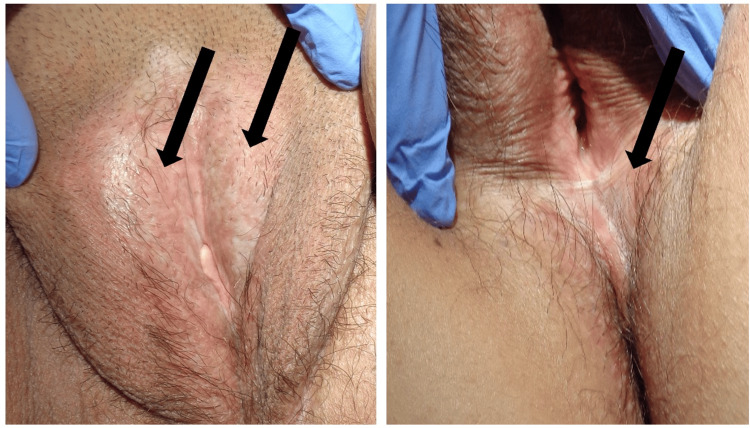
Clinical response to topical steroid ointment. After six months of treatment with topical hydrocortisone butyrate, the lesion exhibited notable pigmentation (black arrows), accompanied by significant improvement in pruritus. Original image by the authors.

The well-defined, sclerotic plaques with epidermal atrophy and dermal homogenization aligned with the features of LS, while the collagen thickening and inflammatory changes in the deeper dermis revealed the features of morphea. Therefore, these histopathological findings suggested a diagnosis of coexisting LS and morphea. Topical hydrocortisone butyrate ointment, commonly used for both LS and morphea, was initiated. During follow-up, mild leukocytosis and elevated CRP levels normalized, likely reflecting the treatment course of transient urinary tract infections. After four months of treatment, the skin lesions showed partial hyperpigmentation, and by six months, most plaques had resolved into hyperpigmented patches (Figure 5).

## Discussion

LS is a chronic inflammatory dermatosis that predominantly affects the vulvar region, presenting as hypopigmented, sclerotic plaques with well-demarcated atrophic changes. Approximately 15% of cases involve extragenital areas, such as the neck, trunk, or proximal extremities [1]. LS is more common in women, with a reported female-to-male ratio ranging from 3:1 to 10:1 [3]. Histopathologically, LS is characterized by hyperkeratosis, epidermal atrophy, basal cell degeneration, and homogenization of collagen fibers in the superficial dermis. Additional findings, such as follicular plugging and lichenoid infiltrates, are distinctive and contribute to differentiating LS from other inflammatory dermatoses. Although the pathogenesis of LS remains unclear, it is thought to involve genetic predisposition, hormonal changes, immune dysregulation, and mechanical or infectious triggers [4,5]. Genital LS carries a higher risk of vulvar squamous cell carcinoma up to 5%, highlighting the importance of long-term surveillance [6].

Distinct from systemic sclerosis, morphea is a localized sclerosing disorder involving collagen proliferation and thickening within the dermis, often extending to deeper tissues. Clinically, it is classified into plaque, linear, and generalized subtypes. Environmental factors, including trauma, infections, radiation exposure, and injections, are considered to act as triggers, activating autoimmune pathways and leading to fibroblast activation and subsequent fibrosis [2]. Morphea lacks systemic features such as digital sclerosis, Raynaud's phenomenon, or internal organ involvement, further distinguishing it from systemic sclerosis [7].

LS and morphea share overlapping clinical and histopathological features, particularly skin sclerosis, which can complicate their differentiation. Chronic phase morphea may extend to the superficial dermis, with increased collagen deposition and edema due to vascular compromise, leading to histopathological features resembling LS [2]. In contrast, chronic course LS may demonstrate collagen thickening in the upper dermis, mimicking morphea [1]. These histopathological overlaps underscore the diagnostic challenges in distinguishing these conditions, particularly when clinical features coexist. These findings suggest that LS and morphea may share common genetic, environmental, and immunological factors, possibly blurring the boundaries between these two conditions.

In a study by Kreuter et al., the features of LS were identified in 6% of 472 morphea patients, including 19 extragenital and eight genital cases [8]. Similarly, Lutz et al. reported that 45% of morphea patients had concurrent genital LS [9], and Farrell et al. found that seven out of nine patients with genital LS also had morphea [10]. Although the coexistence of LS and morphea in distinct lesions within the same patient is well recognized, their occurrence within a single lesion is rare and predominantly observed in extragenital areas [11-18]. Thus, anogenital overlap of these conditions is exceedingly rare. These previous reports highlight the potential interplay between the two conditions and raise questions regarding their classification as distinct entities or varying manifestations within a shared disease spectrum.

In the present case, the vulvar lesion exhibited clinical features consistent with LS but demonstrated histopathological findings characteristic of both LS and morphea. Table 1 summarizes the features of LS and morphea in comparison to this case.

**Table 1:** Clinical and histopathological comparison of lichen sclerosus, morphea, and the current case. This table outlines key similarities and differences among lichen sclerosus, morphea, and the current case, focusing on symptoms, demographics, affected regions, histopathology, and immunological findings.

Parameters	Lichen Sclerosus (LS)	Morphea (Localized Scleroderma)	Current Case
Symptoms	Itching, pain	Minimal or none	Itching, pain
Age/gender	Pre-menarchal and postmenopausal women	Women aged 20–40 years	Elderly female
Affected regions	Predominantly affecting the vulvar region	Commonly affecting the back, chest, and abdomen	Vulvar and perianal regions
Histopathological findings	Epidermal atrophy, liquefactive degeneration, edema in the superficial dermis, homogenization of collagen fibers (Advanced stage: proliferation of collagen fibers in the superficial dermis may occur)	Proliferation and swelling of collagen fibers in the dermis to subcutaneous tissues (Advanced stage: edema in the superficial dermis due to vascular compromise may occur)	Epidermal atrophy, liquefactive degeneration, homogenization of collagen fibers in the superficial dermis, thickening of collagen fibers in the deeper dermis
Immunological abnormalities	None	Positive for antinuclear antibodies, anti-ssDNA antibodies, and anti-histone antibodies	No immunological abnormalities

Although the precise mechanisms underlying the coexistence of LS and morphea remain unclear, their pathogenesis is thought to involve multiple interconnected factors. These include oxidative stress-induced DNA damage, specific human leukocyte antigen (HLA) types associated with genetic predisposition, autoimmune pathways mediated by cytokines, and epigenetic regulation, such as the involvement of microRNA-155 [1,10]. In this case, recurrent urinary tract infections and hormonal fluctuations associated with menopause may play an important role in the development of these co-existing conditions. While cases exhibiting features of both LS and morphea have been reported, the relationship between these diseases remains poorly understood. Further accumulation of cases and studies is required to investigate the potential pathophysiological connections between LS and morphea and ultimately clarify their classification within a shared disease spectrum.

## Conclusions

This case underscores the diagnostic complexities associated with the coexistence of LS and morphea within a single vulvar lesion, a rare presentation that highlights their overlapping clinical and histopathological features. The findings suggest a potential interplay between these conditions, raising the possibility that LS and morphea may represent varying manifestations along a shared disease spectrum. Morphea has the potential to cause deeper inflammation, leading to scar formation and systemic symptoms associated with comorbid autoimmune diseases, thus necessitating differentiation from localized lesions of LS in terms of treatment strategies and prognosis. Further research is warranted to elucidate their pathogenesis and develop more precise diagnostic criteria and management strategies.
